# Supplementary material for: Identification of Unique microRNA Profiles in Different Types of Idiopathic Inflammatory Myopathy
Source: Cells. 2023 Sep 2;12(17):2198. doi: 10.3390/cells12172198 (PMC10487266; doi:10.3390/cells12172198)
Supplement: Supplementary file 1 [file cells-12-02198-s001.zip › cells-2535552-supplementary.pdf]

Supplementary Materials

Supplementary methods:

Quality control assessment of nCounter data and data preparation for the differential expression analysis

We first evaluated the performance of the Nanostring assay according to the metrics of the company and using the pipeline designed by Bhattacharya et al. (referenced in the main manuscript). We also visualized the raw counts as part of the quality control assessment and we noticed that one of the negative controls, negative control B, presented a higher expression in comparison to the other negative controls. To investigate the possibility that the signal detected for negative control B was incorrectly assigned from that of a different probe, we calculated the correlation between the raw counts of negative control B and the rest of the probes from the panel. We found that miR-206 had an almost perfect correlation ($r_s = 0.98$, p value $=1.4 \times 10^{-32}$), so we thought it was possible that the counts detected for this negative control were due to a cross-detection of miR-206. Supporting this theory, we observed that the reporter probe for the negative control B was very similar to that of miR-206 (YRGBYR and YRGBYB, respectively), differing in only the last of the six fluorochrome sequence. We also detected a high correlation between miR-206 counts and the counts for other probes having at least an identical sequence of four fluorochromes in the reporter probe sequences (miR-1269b, miR-378c, miR-4532). Cross detection events involving miR-206 could be favored by the much higher expression of miR-206 relative to negative control B, miR-1269b, miR-378c and miR-4532 (817, 883, 2716, and 2470 times higher median expression, respectively).

This unexpected event prompted us to systematically assess the possibility that similar cross-detection events might have occurred for other probes whose number of counts could have been wrongly assigned due to interferences with the detection of remarkably highly expressed miRNAs. We identified pairs of probes that had a correlation above 0.85 between their counts and an identical sequence of four fluorophores in their reporter probe, and we excluded from the analysis the probe with lower expression if the median expressions of the two probes differed at least 100 times. This resulted in 21 probes excluded from our analysis. We also scrutinized the sequence of the probes that, according to the manufacturer, can detect the expression of more than one mature miRNA and we excluded from the analysis the probes whose sequence did not match the canonical sequence of all the miRNAs claimed to be detected by the probe, annotated in miRBase. In total, 13 probes were excluded based on the specificity of the probe.

The mRNA reference controls included in the assay from Nanostring are not appropriate for the normalization of miRNAs, and thus we added another modification to the pipeline for the analysis of Nanostring data proposed by Bhattacharya et al. We decided to use as reference for the normalization the miRNAs whose number of counts was above the limit of detection (LOD) in every sample. To calculate the LOD, we modified the pipeline designed by Bhattacharya et al. for the analysis of Nanostring mRNA data to consider the controls that Nanostring includes for the ligation step, specific to the miRNA assay, in addition to the hybridization negative controls (except negative control B, due to the cross-detection mentioned above). The LOD was defined as the mean of counts of the negative controls plus two standard deviations. Following Bhattacharya et al. pipeline, we excluded as reference miRNAs those that were potentially differentially expressed for any comparison. Forty-one probes met the conditions to be considered as reference for normalization.

For the analysis, we used the probes whose number of raw counts were above the LOD in at least the majority samples of one of the groups included in the study (classified based on the positivity for each autoantibody or as IBM or NT). The number of counts was also assessed after the differential expression analysis to confirm that the miRNAs identified as differentially expressed for one group relative to the other in a particular

comparison presented an expression above the LOD in most of the samples of the group with the higher expression.

Supplementary tables

Table S1. Demographic and clinical features of the study subjects.

	NT (n=6)	IBM (n=6)	IMNM (n=12)	DM (n=18)	AS (n=6)
**Autoantibody**					
anti-Jo1	0% (0)	0% (0)	0% (0)	0% (0)	100% (6)
anti-HMGCR	0% (0)	0% (0)	50% (6)	0% (0)	0% (0)
anti-SRP	0% (0)	0% (0)	50% (6)	0% (0)	0% (0)
anti-MDA5	0% (0)	0% (0)	0% (0)	11.11% (2)	0% (0)
anti-Mi2	0% (0)	0% (0)	0% (0)	27.78% (5)	0% (0)
anti-NXP2	0% (0)	0% (0)	0% (0)	27.78% (5)	0% (0)
anti-TIF1	0% (0)	0% (0)	0% (0)	33.33% (6)	0% (0)
**Gender**					
Female	66.67% (4)	33.33% (2)	83.33% (10)	55.56% (10)	83.33% (5)
Male	33.33% (2)	66.67% (4)	16.67% (2)	44.44% (8)	16.67% (1)
**Race**					
Black	0% (0)	0% (0)	25% (3)	11.11% (2)	50% (3)
White	83.33% (5)	100% (6)	66.67% (8)	77.78% (14)	33.33% (2)
Other	16.67% (1)	0% (0)	8.33% (1)	11.11% (2)	16.67% (1)
**Age at biopsy**	42.91 ± 10.88	63.20 ± 10.71	51.45 ± 17.04	57.95 ± 12.53	46.54 ± 13.46
**Years from onset**	0	7.5 ± 6.36	2.26 ± 2.36	0.82 ± 1.40	1.38 ± 1.86
**Treatments**					
Corticosteroids	0% (0)	33.33% (2)	41.67% (5)	66.67% (12)	66.67% (4)
Azathioprine	0% (0)	0% (0)	0% (0)	5.56% (1)	16.67% (1)
Methotrexate	0% (0)	33.33% (2)	25% (3)	22.22% (4)	33.33% (2)
Mycophenolate	0% (0)	16.67% (1)	8.33% (1)	16.67% (3)	16.67% (1)
IVIG	0% (0)	0% (0)	8.33% (1)	5.56% (1)	0% (0)
Cyclosporine	0% (0)	0% (0)	0% (0)	5.56% (1)	0% (0)
Any immunosuppressant	0% (0)	66.67% (4)	41.67% (5)	66.67% (12)	66.67% (4)
**Localization biopsy**					
Upper extermity (biceps or deltoid)	66.67% (4)	50% (3)	41.66% (5)	55.56% (10)	33.33% (2)
Lower extremity (quadriceps)	33.33% (2)	50% (3)	58.33% (7)	44.44% (8)	66.67% (4)
**Biopsy features**					
Perifascicular atrophy	0% (0)	0% (0)	8.33% (1)	72.22% (13)	33.33% (2)
Perivascular inflammation	0% (0)	33.33% (2)	16.67% (2)	66.67% (12)	33.33% (2)
Myofiber necrosis	0% (0)	83.33% (5)	83.33% (10)	72.22% (13)	66.67% (4)
Myofiber regeneration	0% (0)	100% (6)	75% (9)	50% (9)	83.33% (5)
Primary inflammation (invasion)	0% (0)	66.67% (4)	0% (0)	0% (0)	16.67% (1)
Rimmed vacuoles	0% (0)	100% (6)	0% (0)	0% (0)	0% (0)
None of the above	100% (6)	0% (0)	8.33% (1)	16.67% (3)	16.67% (1)

Supplementary figures:

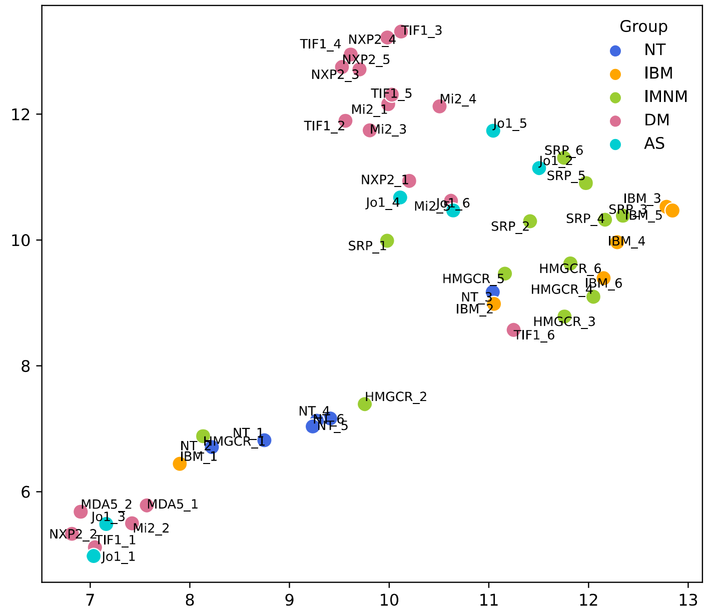

Figure S1. UMAP of the samples. UMAP (Uniform Manifold Approximation and Projection) representation of the samples using the data of the 36 miRNAs that were differentially expressed compared to controls in one or more types of IIM. The UMAP python package (v.0.5.3) was used for dimensionality reduction of the data.

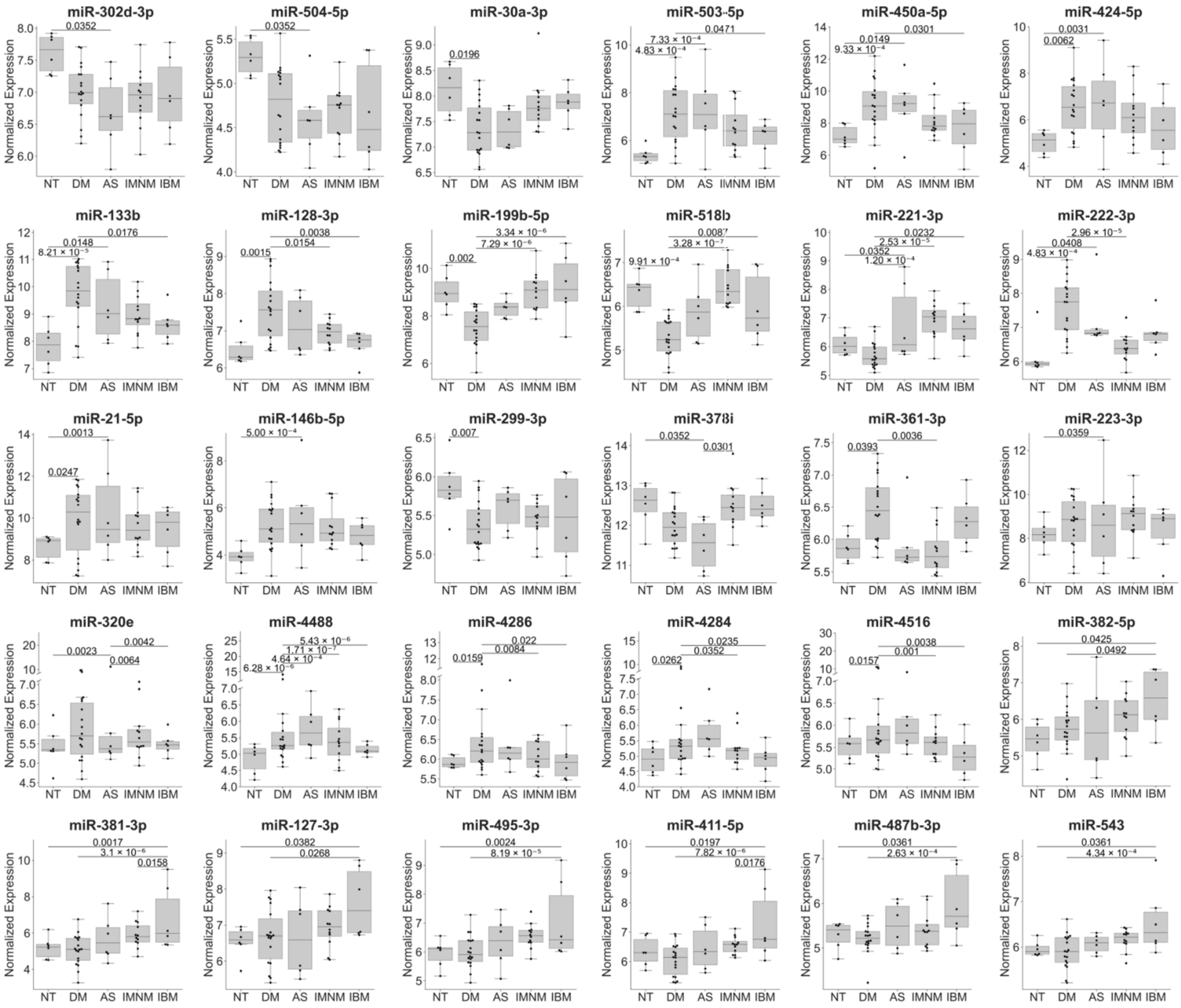

Figure S2. Expression profile of differentially expressed miRNAs. Log-scaled normalized expression levels of miRNAs that are differentially expressed in at least one type of IIM and that are not included in Figure 1c in the main text. Note the y-axis break in some graphs to better visualize different ranges of logarithmic normalized expression levels of miRNAs that present exceptionally high expression in a few samples.

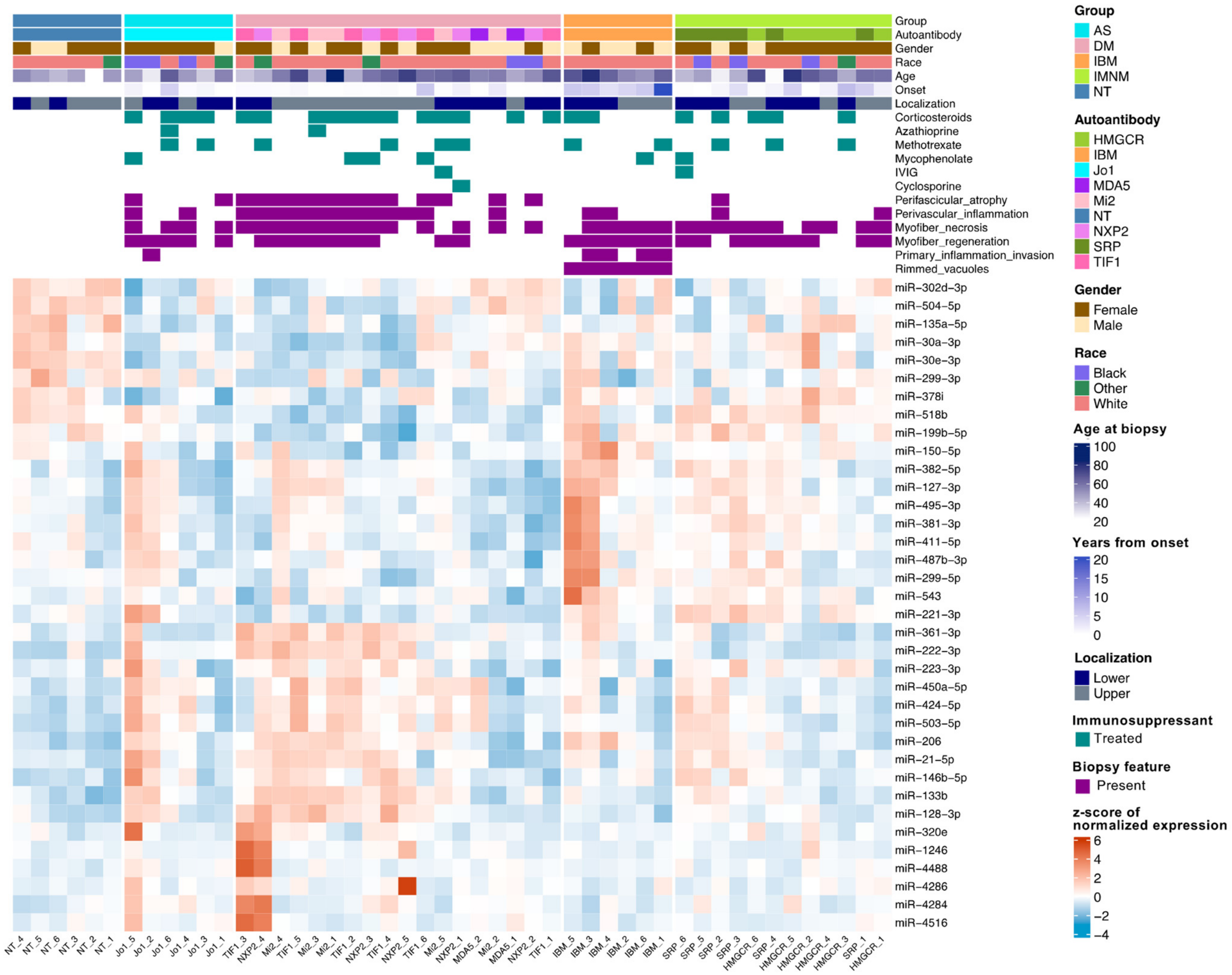

Figure S3. Heatmap of differentially expressed miRNAs with corresponding demographic, clinical, and histopathologic information. Heatmap representation of the normalized (z-score) expression data of the 36 miRNAs that were differentially expressed compared to controls in one or more types of IIM. The heatmap was depicted using the complex heatmap R package (v.2.10.0).

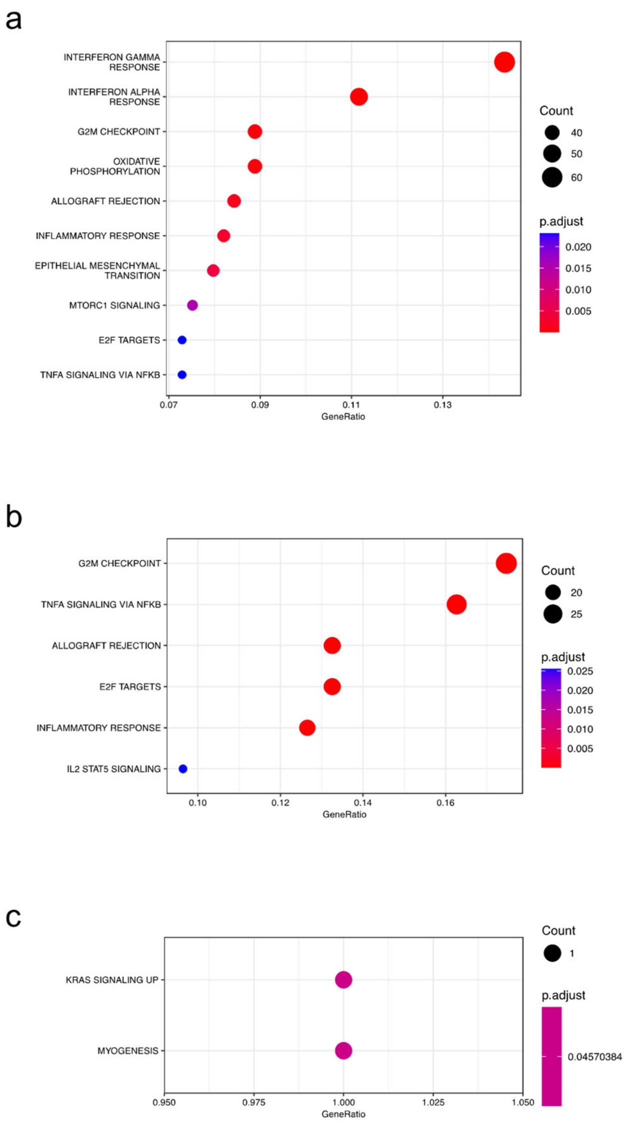

Figure S4. Pathway enrichment analysis of selected targets of the differentially expressed miRNAs. Dot plots represent the results of the pathway enrichment analysis for the mRNAs that are differentially expressed in a type of IIM and that in the same IIM type have a significative correlated expression with targeting miRNAs that are also differentially expressed in DM (**a**), AS (**b**) or IBM (**c**).

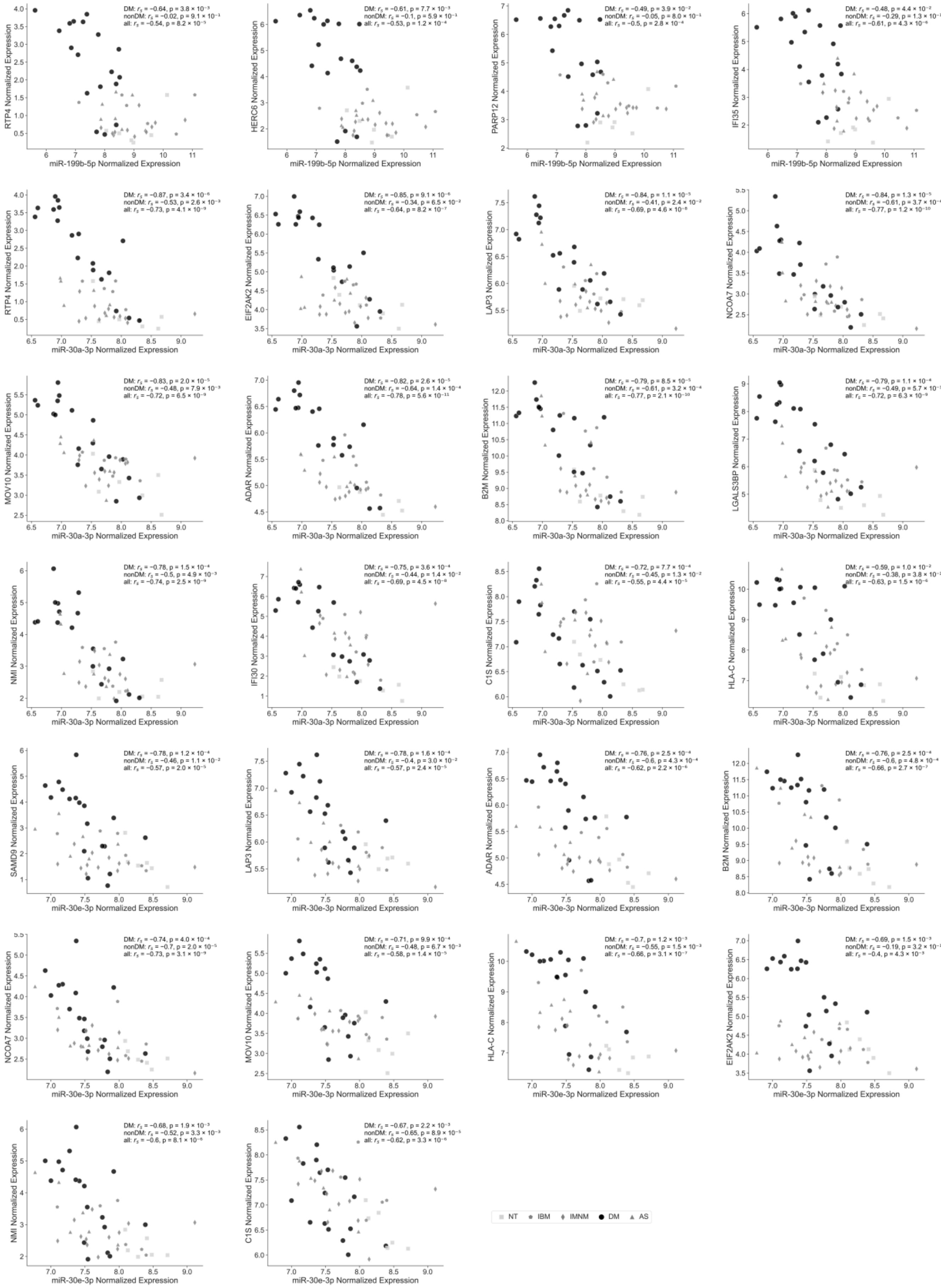

Figure S5. Correlation of miR-30a-3p, miR-30e-3p, and miR-199b-5p with DM differentially expressed targets that are associated with the interferon-alpha response. Scatterplots represent the

negative correlations of miR-30a-3p, miR-30e-3p, and miR-199b-5p with their DM differentially expressed targets that are involved in the interferon-alpha response. The miRNA-mRNA correlation of the pairs with the strongest inverse correlation for each of the three miRNAs is shown in Figure 3b in the main text. Spearman's rank correlation (r_s) and *p* values of the correlation across DM samples, all samples excluding DM samples (named nonDM for short), or the total of samples (all), are shown.
